# Huperzine A Ameliorates Cognitive Deficits in Streptozotocin-Induced Diabetic Rats

**DOI:** 10.3390/ijms15057667

**Published:** 2014-05-05

**Authors:** Xiao-Yuan Mao, Dan-Feng Cao, Xi Li, Ji-Ye Yin, Zhi-Bin Wang, Ying Zhang, Chen-Xue Mao, Hong-Hao Zhou, Zhao-Qian Liu

**Affiliations:** 1 Institute of Clinical Pharmacology, Hunan Key Laboratory of Pharmacogenetics, Central South University, Changsha 410078, China; 2 Institute of Clinical Pharmacology, Xiangya Hospital, Central South University, Changsha 410008, China; 3 Department of Genetics, Institute of Medical Biology, Chinese Academy of Medical Sciences & Peking Union Medical College, Kunming 650118, China; 4 School of Life Sciences, Zhengzhou University, Zhengzhou 450001, China

**Keywords:** huperzine A, diabetes-associated cognitive decline, brain-derived neurotrophic factor, oxidative stress, inflammation, apoptosis

## Abstract

The present study was designed to probe the effects of Huperzine A (HupA) on diabetes-associated cognitive decline (DACD) using a streptozotocin (STZ)-injected rat model. Diabetic rats were treated with HupA (0.05 and 0.1 mg/kg) for seven weeks. Memory functions were evaluated by the water maze test. Nissl staining was selected for detecting neuronal loss. Protein and mRNA levels of brain-derived neurotrophic factor (BDNF) were analyzed by ELISA and real-time PCR, respectively. The activities of choline acetylase (ChAT), Acetylcholinesterase (AChE), malondialdehyde (MDA), superoxide dismutase (SOD), glutathione peroxidase (GSH-Px), catalase (CAT), NF-κB p65 unit, TNF-α, IL-1β, IL-6 and caspase-3 were measured using corresponding kits. After seven weeks, diabetic rats exhibited remarkable reductions in: body weight, percentage of time spent in target quadrant, number of times crossing the platform, ChAT and BDNF levels, SOD, GSH-Px and CAT accompanied with increases in neuronal damage, plasma glucose levels, escape latency, mean path length, AChE, MDA level as well as CAT, NF-κB p65 unit, TNF-α, IL-1β, IL-6 and caspase-3 in cerebral cortex and hippocampus. Supplementation with HupA significantly and dose-dependently reversed the corresponding values in diabetes. It is concluded that HupA ameliorates DACD via modulating BDNF, oxidative stress, inflammation and apoptosis.

## 1. Introduction

Emerging evidence has demonstrated that diabetes mellitus (DM), a chronic metabolic disorder, can cause serious neuronal impairment and cognitive deficits, which are conceived of as the most common symptom [1,2]. It was previously reported that patients with DM had an approximate double risk for the generation of dementia in a perspective population based study of 6370 elderly individuals [3]. In 2006, Mijnhout *et al.* [4] proposed a new concept “diabetes-associated cognitive decline (DACD)” in diabetes research. This concept emphasizes the importance of diabetes-induced cognitive impairment. It is of desperate need to figure out the pathogenesis of DACD and further explore the potential therapeutic approach for the prevention of these cognitive symptoms.

The pathogenesis of cognitive dysfunction seems to be a multifactorial process under diabetic circumstances [5]. Substantial evidence supports the notion that long-term metabolic control and microvascular factors are closely correlated with cognitive impairment caused by diabetes [6,7]. Prior work disclosed that hyperglycemia-associated microvascular changes in the brain could induce cognitive injury in patients with type 1 diabetes (T1DM), and intensive therapy with insulin in T1DM has remarkably decreased the risk of microvascular and macrovascular complications [6]. The crucial role of hyperglycemia in the generation of cognitive decline in patients with T1DM, implies that drugs that improve glycemic control may be useful for the amelioration of DACD.

Brain-derived neurotrophic factor (BDNF) is one of the most important neurotrophic factors that regulates survival, differentiation and maintenance of function in diverse neuronal populations [8]. In addition, BDNF is also recognized as a key component of the hypothalamic pathway that controls body weight and energy homeostasis [9]. Administration of BDNF in the brain was found to remarkably reduce food intake, increase energy expenditure and alleviate hyperinsulinaemia and hyperglycemia in diabetic db/db mice [10]. More importantly, a previous investigation illustrated that decreased levels of BDNF in hippocampus contributed to the impaired cognitive performance in streptozotocin (STZ)-induced diabetic rats [11]. Taking into account the fact that BDNF is closely linked with cognition, it is of great interest to explore the neuroprotective agents that may attenuate the cognitive decline due to diabetes.

Previous studies have shown that oxidative stress is involved in the pathogenesis of the diabetic state [12,13]. The increased oxidative stress under diabetic conditions produces oxidative damage in different regions of the brain including the hippocampus and the cerebral cortex. Additionally, excessive production of oxygen-free radicals and/or antioxidant deficiency to various brain regions were reported to cause morphological abnormalities and memory deficits during aging [14]. Treatment with antioxidants has been shown to protect neurons against diabetes-induced excitotoxicity and neurodegenerative conditions [15]. Additionally, diabetes is also accompanied with the marked release of inflammatory cytokines [13,16]. A recent report demonstrated that the activated nuclear transcription factor κB (NF-κB) signaling pathway led to diabetes-induced cognitive impairment [17].

Huperzine A (HupA), a novel *Lycopodium alkaloid* extracted from the Chinese folk medicine *Huperzia serrate*, has been demonstrated to effectively improve cognitive function after transient global ischemia in gerbils [18] and in Alzheimer’s disease [19]. It has also been reported that HupA can attenuate the impairment of cognitive deficits following cerebral ischemia and reperfusion in mice via promoting BDNF production [20]. Recently, HupA was found to exert protection against oxidative stress damage in SH-SY5Y neuroblastoma cells [21]. However, whether HupA has a neuroprotective role in DACD remains elusive. Furthermore, as BDNF and oxidative stress play a critical role in learning and memory functions, we speculate that they are involved in HupA’s neuroprotection. The present study was designed to clarify the neuroprotective effect of HupA against DACD using a rat model of diabetes and whether the neuroprotection mechanism involves BDNF and oxidative stress.

## 2. Results

### 2.1. Effects of HupA on Body Weight and Blood Glucose Levels

As shown in Table 1 a significant reduction of body weight was observed in diabetic rats (*p* < 0.01), in comparison to the control group. However, HupA treatment with doses of 0.05 and 0.1 mg/kg in diabetic animals remarkably reversed the alteration (*p* < 0.01), compared to the vehicle group. In addition, there was an obvious decrease of plasma glucose level in the diabetes-induced group (*p* < 0.01) compared to the age-matched control. Administration of HupA with different doses (0.05 and 0.1 mg/kg) could improve the glucose level in STZ-treated animals (*p* < 0.01).

### 2.2. Effects of HupA on Diabetes-Induced Cognitive Deficit

**Figure 1.**
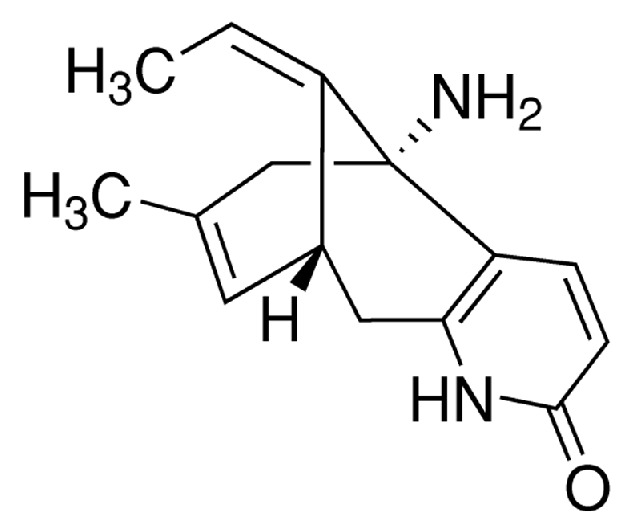
The chemical structure of HupA [22].

**Figure 2.**
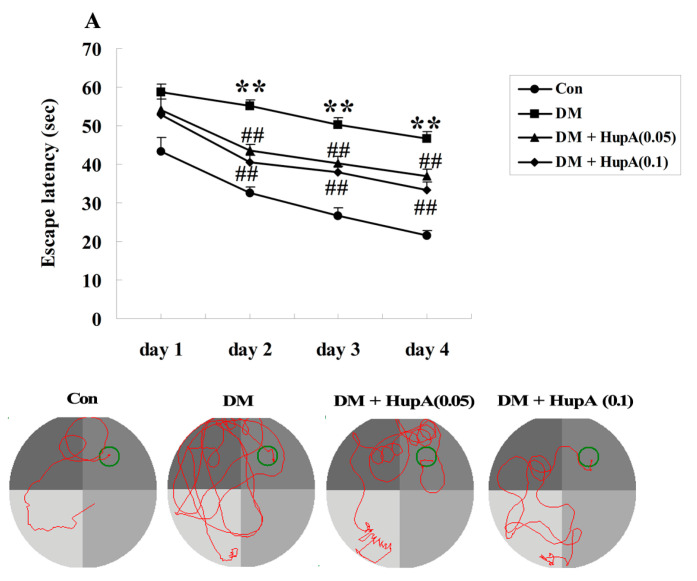

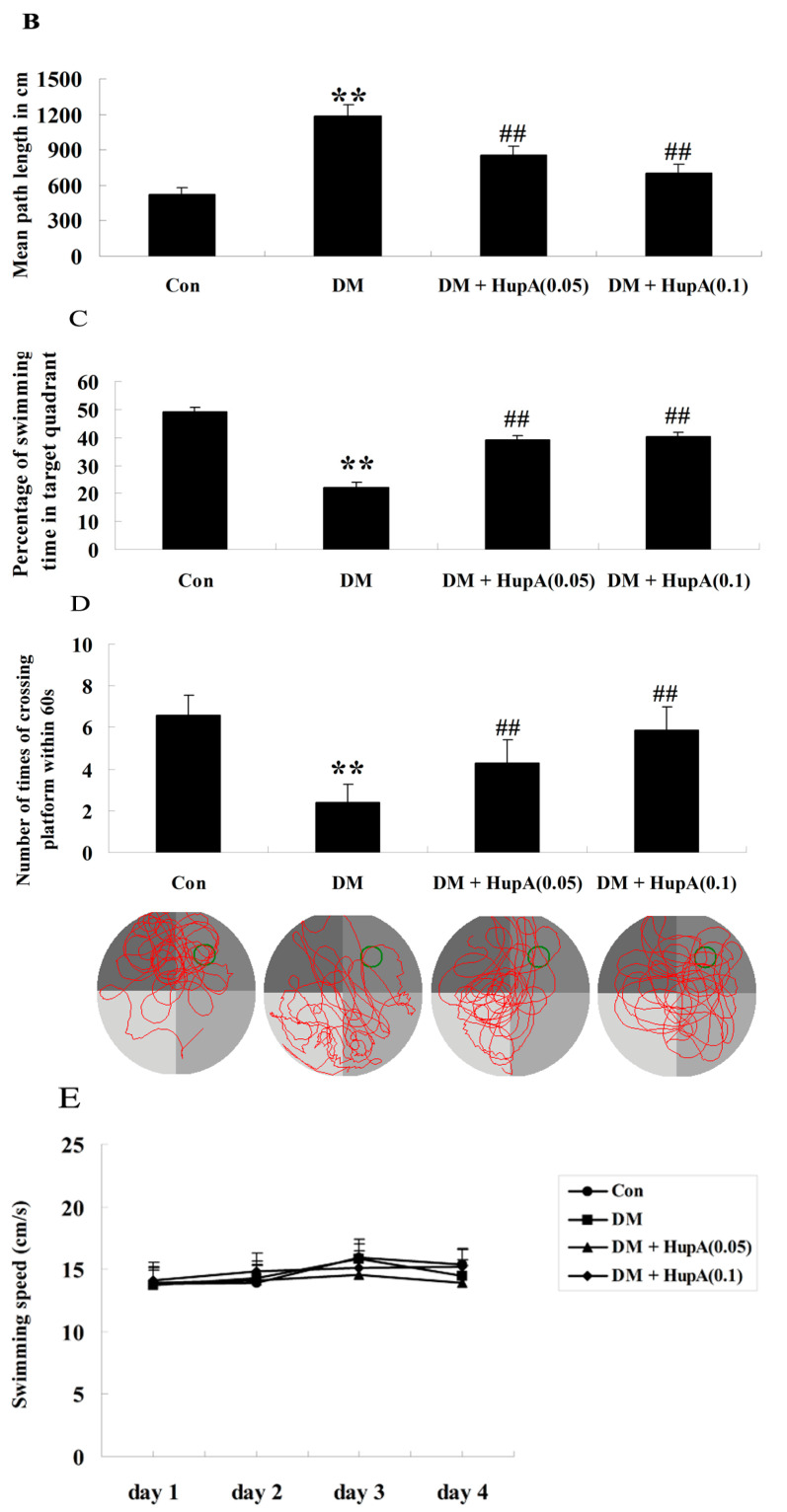
Effects of HupA on the escape latency (**A**); mean path length (**B**); mean percentage of time spent in the target quadrant (**C**); the number of times of crossing platform (**D**) and swimming speed (**E**) in control and diabetic rats (*n* = 8, mean ± S.D.). ** *p* < 0.01 compared with Con group; ^##^ *p* < 0.01 compared with DM group. Con, control; DM, diabetes; DM + HupA (0.05), huperzine A (0.05 mg/kg)-treated; DM + HupA (0.1), huperzine A (0.1 mg/kg)-treated groups.

The chemical structure of HupA was displayed in Figure 1. After seven weeks of HupA or vehicle treatment, the Morris water maze task was performed to assess the learning and memory performance in different groups. The mean escape latency was firstly analyzed in our present investigation, as shown in Figure 2A. It was noteworthy that no significant difference was found between any of the groups on the first day. Nevertheless, from the second day onwards, diabetic rats exhibited longer transfer latency (*p* < 0.01), than the control group. The poorer performance was evidently suppressed in STZ-induced rats treated with HupA by the doses of 0.05 and 0.1 mg/kg. Likewise, Figure 2B also illuminated a marked elevation of mean path length in diabetes-induced animals for four training days (*p* < 0.01) compared to the controls. Chronic supplement with HupA dose-dependently decreased this index (*p* < 0.01), in comparison with the vehicle group. Furthermore, there were obvious reductions of time spent in the target quadrant and the number of times the animals crossed the former platform location in diabetic rats (*p* < 0.01), when compared with the control group. However, the two values were significantly diminished after treating with HupA, as displayed in Figure 2C,D. In addition, the swimming speed was also detected and we found no statistical significance among different groups during four days of training, as illustrated in Figure 2E.

### 2.3. Effects of HupA on the Hippocampal Neuronal Loss in Diabetic Rats

**Figure 3.**
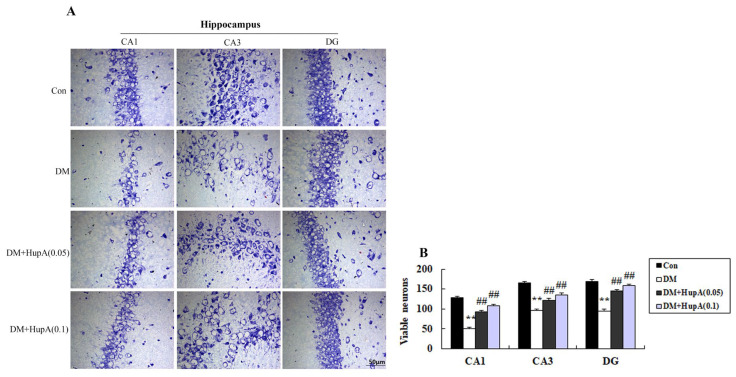
Effects of HupA on the hippocampal neuronal loss control and diabetic rats. (**A**) and (**B**) were the representative photographs of hippocampal neuronal loss and quantitative analysis of viable neurons, respectively. ** *p* < 0.01 compared with Con group; ^##^ *p* < 0.01 compared with DM group. Con, control; DM, diabetes; DM + HupA (0.05), huperzine A (0.05 mg/kg)-treated; DM + HupA (0.1), huperzine A (0.1 mg/kg)-treated groups. Scale bar: 50 μm.

The neuronal loss was evaluated by Nissl staining. As shown in Figure 3, diabetic rats exhibited marked neuronal death in hippocampal CA1, CA3 and DG regions. However, HupA in doses of 0.05 and 0.1 mg/kg significantly alleviated neuronal damage in the selected subregions of hippocampus including CA1, CA3 and DG.

### 2.4. Effects of HupA on the Activities of AChE and ChAT in Diabetic Rats Brain

**Figure 4.**
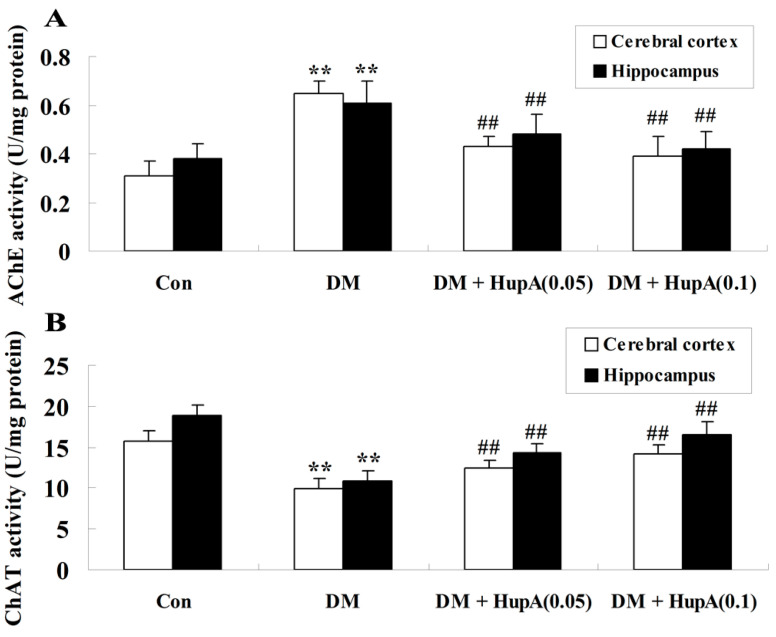
Effects of HupA on the activities of AChE (**A**) and ChAT (**B**) in cerebral cortex and hippocampus of control and diabetic rats (*n* = 8, mean ± S.D.). ** *p* < 0.01 compared with Con group; ^##^ *p* < 0.01 compared with DM group. Con, control; DM, diabetes; DM + HupA (0.05), huperzine A (0.05 mg/kg)-treated; DM + HupA (0.1), huperzine A (0.1 mg/kg)-treated groups.

To explore whether HupA could influence the activities of AChE and ChAT in diabetes, cerebral cortex and hippocampus samples were separated from different groups and subjected to the colorimetric tests in our present work. As shown in Figure 4A, there was a marked elevation of AChE activity in the diabetic group (*p* < 0.01), as compared to the controls. This phenomenon was obviously reversed after HupA treatment. In contrast, the level of ChAT was decreased in diabetic rats and HupA treatment obviously increased the ChAT activity in a dose-dependent manner (Figure 4B).

### 2.5. Effects of HupA on mRNA and Protein Levels of BDNF in Diabetic Rat Brain

**Figure 5.**
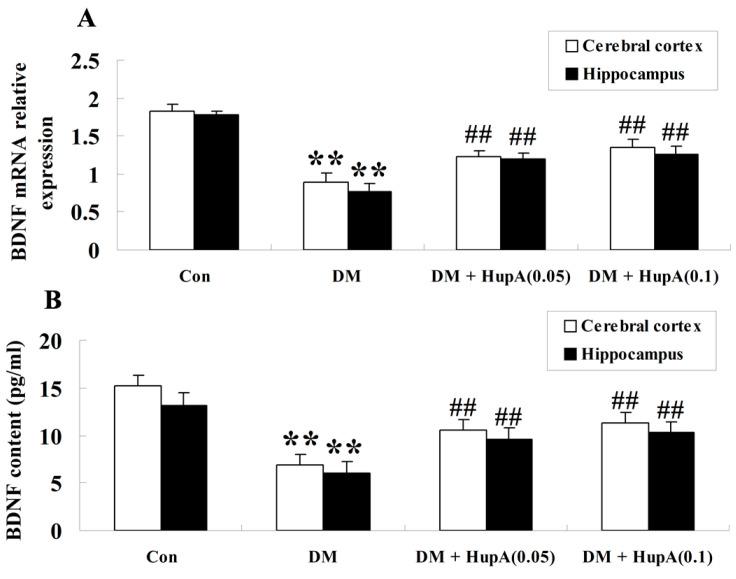
Effects of HupA on the mRNA (**A**) and protein levels (**B**) of BDNF in cerebral cortex and hippocampus of control and diabetic rats (*n* = 8, mean ± S.D.). ** *p* < 0.01 compared with Con group; ^##^ *p* < 0.01 compared with DM group. Con, control; DM, diabetes; DM + HupA (0.05), huperzine A (0.05 mg/kg)-treated; DM + HupA (0.1), huperzine A (0.1 mg/kg)-treated groups.

For analyzing the mRNA expression level of BDNF from different groups, a SYBR Green I fluorescent real-time PCR approach was employed to detect the cDNA-amplified products in cerebral cortex and hippocampus of rats after seven weeks of HupA or vehicle treatment. Figure 5A revealed that the mRNA level of BDNF was statistically downregulated both in cerebral cortex and the hippocampus of diabetic rat brain (*p* < 0.01) when compared to the controls. HupA treatment with doses of 0.05 and 0.1 mg/kg both produced a statistical increase of BDNF at the mRNA level. The effects of HupA on BDNF expression were corroborated by BDNF immunoassay. Diabetic rats exhibited a lower protein level of BDNF, but this was remarkably enhanced in the cerebral cortex and hippocampus by different doses of HupA (Figure 5B).

### 2.6. Effects of HupA on Oxidative Stress in Diabetic Rat Brain

**Figure 6.**
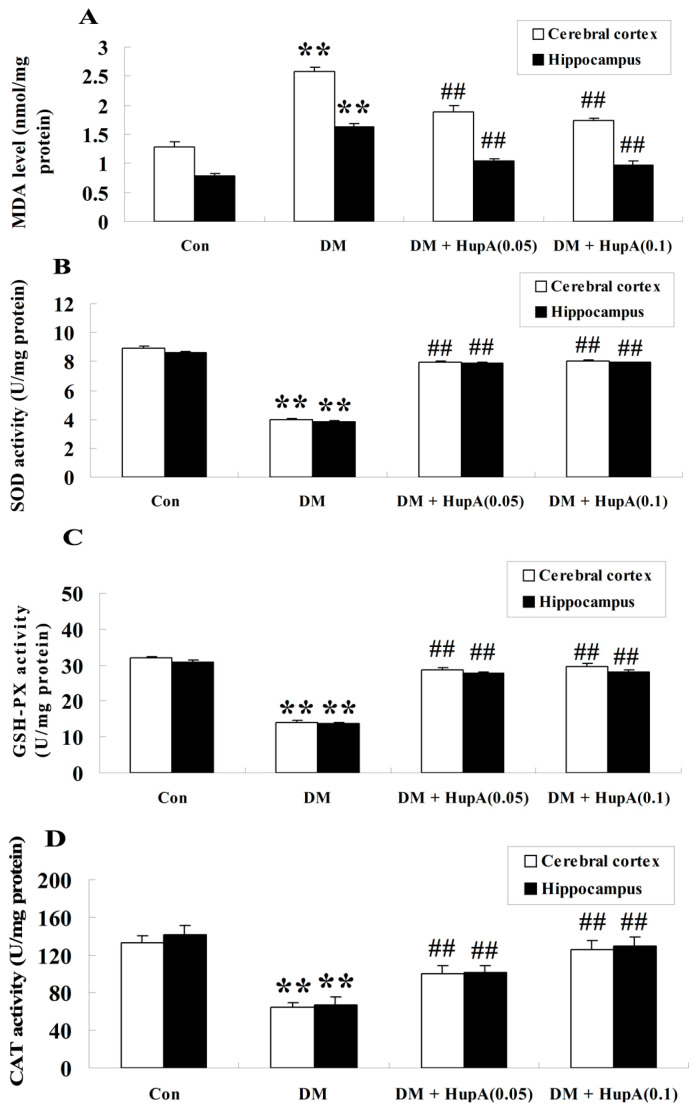
Effects of HupA on the oxidative stress in cerebral cortex and hippocampus of control and diabetic rats (*n* = 8, mean ± S.D.). (**A**–**D**) showed the oxidative production of MDA, SOD, GSH-PX and CAT activities, respectively in the cerebral cortex and hippocampus of control and diabetic rat brain. ** *p* < 0.01 compared with Con group; ^##^ *p* < 0.01 compared with DM group. Con, control; DM, diabetes; DM + HupA (0.05), huperzine A (0.05 mg/kg)-treated; DM + HupA (0.1), huperzine A (0.1 mg/kg)-treated groups.

We subsequently investigated whether HupA influenced oxidative stress in the cerebral cortex and hippocampus of diabetic rat brain by detecting the level of MDA and anti-oxidant enzymes (SOD, GSH-PX and CAT). It was noted that STZ-induced diabetes markedly increased the oxidative production of MDA both in cerebral cortex and hippocampus (*p* < 0.01), compared to the age-matched control group (Figure 6A). Nevertheless, 7-week HupA statistically inhibited the content of MDA in diabetic rat brain. Additionally, the activities of all detected anti-oxidant enzymes including SOD, GSH-PX and CAT were significantly diminished in diabetes-induced rats (*p* < 0.01) compared with controls (Figure 6B–D). Chronic treatment with HupA dose-dependently augmented these values both in the cerebral cortex and hippocampus of diabetic rat brain.

### 2.7. Effects of HupA on Inflammatory Cytokines in Diabetic Rat Brain

**Figure 7.**
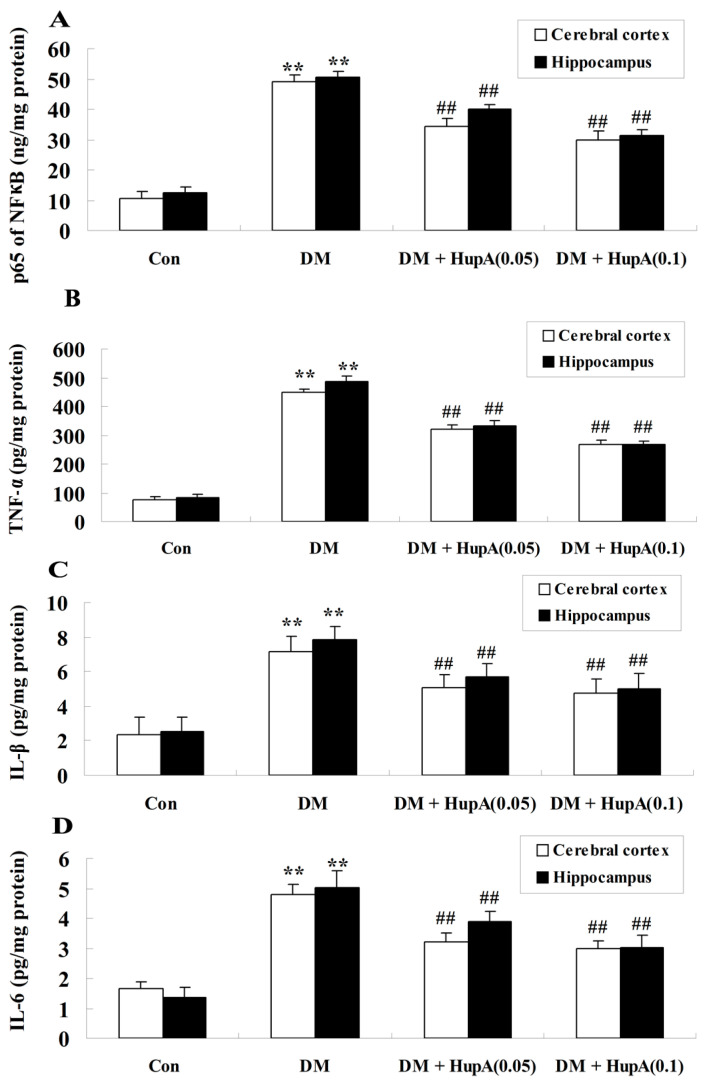
Effects of HupA on the inflammatory cytokines in cerebral cortex and hippocampus of control and diabetic rats (*n* = 8, mean ± S.D.). (**A**–**D**) displayed NF κB p65 subunit, TNF-α, IL-1β and IL-6 levels in the cerebral cortex and hippocampus of control and diabetic rat brain. ** *p* < 0.01 compared with Con group; ^##^ *p* < 0.01 compared with DM group. Con, control; DM, diabetes; DM + HupA (0.05), huperzine A (0.05 mg/kg)-treated; DM + HupA (0.1), huperzine A (0.1 mg/kg)-treated groups.

The effects of HupA on the inflammation induced by diabetes were also detected in our present study. It is interesting that the activities of major inflammatory factors including NF-κB, TNF-α, IL-1β and IL-6 were found to be notably elevated in the cerebral cortex and hippocampus of STZ-injected rats (*p* < 0.01), compared to those of the control group (Figure 7A–D). HupA (0.05 and 0.1 mg/kg) treatment markedly and dose-dependently suppressed inflammatory responses in different regions of diabetic rat brain.

### 2.8. Effects of HupA on Caspase-3 Activity in Diabetic Rat Brain

**Figure 8.**
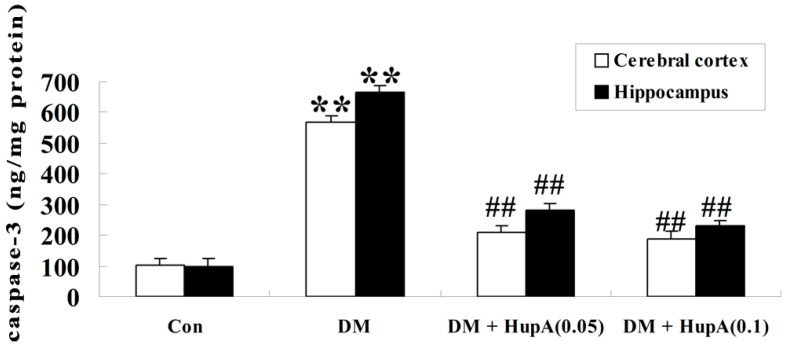
Effects of HupA on the caspase-3 activity in cerebral cortex and hippocampus of control and diabetic rats (*n* = 8, mean ± S.D.). ** *p* < 0.01 compared with Con group; ^##^ *p* < 0.01 compared with DM group. Con, control; DM, diabetes; DM + HupA (0.05), huperzine A (0.05 mg/kg)-treated; DM + HupA (0.1), huperzine A (0.1 mg/kg)-treated groups.

As illustrated in Figure 8, caspase-3 levels were found to be obviously augmented both in cerebral cortex and hippocampus of diabetic rat brain (*p* < 0.01), in comparison to the control group. However, treatment with HupA by different doses (0.05 and 0.1 mg/kg) significantly caused a greater attenuation of caspase-3 activity in the selected regions of STZ-injected rats (*p* < 0.01).

## 3. Discussion

The major findings of the current study illustrated that HupA attenuated DACD in rats. And its neuroprotection might be associated with enhancement of BDNF expression, inhibition of oxidative stress and suppression of inflammation.

It was previously reported that hyperglycemia could serve as a determinant of cognitive decline in patients with type 1 diabetes [6], implicating that glycemic control was imperative for ameliorating diabetes-induced cognitive deficits. In our present work, HupA treatment markedly improved the plasma glucose level of diabetic rats, which suggested that HupA could be used as a good hypoglycemic reagent. Therefore, the good blood glucose control of HupA facilitates its alleviation of cognitive damage induced by diabetes.

Cholinergic dysfunction has been involved in the occurrence of learning and memory deficits caused by diabetic rats [11]. Under normal condition, AChE and ChAT activities are two specific markers of cholinergic neurons in the cerebral cortex and hippocampus. They play a critical role in the modulation of the cholinergic pathway. Results from our current investigation illustrated that chronic treatment with HupA evidently augmented ChAT activity and decreased AChE activity in diabetic rats. A previous report illuminated that HupA had the obvious inhibition of AChE activity and activation of ChAT activity in an Alzheimer transgenic mouse model [22], which was in line with our findings. In fact, our preliminary report found synergetic effects of decreasing plasma glucose level after treating with donepezil (a positive drug for AChE inhition) and HupA (data not shown), indicating that HupA reduced plasma glucose via an AChE-dependent pathway.

BDNF level could diminish the progression of patients with mild cognitive deficits [23] and decreased BDNF was previously reported to exacerbate hippocampal damage during DACD [11]. In addition, the positive correlation with BDNF production and attenuation of cognitive decline exemplified that HupA treatment augmented BDNF content and subsequently attenuated cognitive impairment in a mice model with cerebral ischemia-reperfusion injury. Consistently, our present study disclosed the elevation of BDNF expression at the mRNA and protein levels accompanied with attenuation of cognition in STZ-induced diabetes after treating with HupA, suggesting that HupA ameliorated diabetes-induced cognitive dysfunction, at least, in part by virtue of increasing BDNF expression.

Oxidative stress has been implicated in the generation of d-galactose-induced aging rats which exhibits the characteristics of memory abnormality [24]. It is well known that oxidative stress always leads to oxidative damage of biomacromolecules, including lipoprotein within the cellular membranes. Elevated MDA is regarded as a specific indicator of lipid peroxidation during oxidative impairment [25]. In addition, oxidative injury could also destroy the anti-oxidant defense system, such SOD, GSH-PX and CAT. In fact, it was previously found that oxidative brain damage caused by oxidative stress contributed to the serious impairment of learning and memory deficits during aging in rats [14]. Data from our current work illustrated that oxidative injury (increased MDA content, decreased SOD, GSH-PX and CAT activities) was observed in diabetic rats with cognitive disturbance and the phenomenon was significantly reversed after HupA treatment. Similarly, a previous investigation revealed that HupA could diminish the production of MDA and augment the activities of anti-oxidant enzymes including GSH-PX and CAT in an APP/PS1 mouse model of Alzheimer’s disease [22]. Collectively, these findings suggest that HupA could effectively inhibit oxidative stress induced by DACD in rats.

Pro-inflammatory cytokines play a critical role in the occurrence of cognition-associated neuropathological states. Cumulative evidence demonstrated that the marked release of inflammatory mediators were observed in diabetes-induced cognitive decline of rats [13,16]. Indeed, TNF-α was shown to inhibit the development of long term potentiation in the dentate gyrus subregion of the rat hippocampus [26]. NF-κB might also be conceived of as one of the critical inflammatory response factors and the p65 subunit is positively correlated with the NF-κB signaling pathway [27]. It was previously reported that TNF-α, IL-1β and IL-6 were involved in the modulation of NF-κB signaling and caspase-3 activation in the diabetic rat brain [28]. Our current investigations indicated that HupA significantly and dose-dependently suppressed NF-κB signaling via inhibition of inflammation. A previous investigation revealed that HupA inhibited the overexpression of TNF-α and improved the cognitive decline in a rat model of cerebral hypoperfusion, which was, in part, in agreement with our current findings [29].

Oxidative stress could seriously contribute to mitochondrial dysfunction and ultimately trigger a series of caspase-activated apoptotic cascades in neurons [30]. Caspase-3 is regarded as an indicator of apoptosis. Our present investigation showed the remarkable elevation of caspase-3 activity in the cerebral cortex and hippocampus of diabetic rat brain and this effect was blocked by HupA treatment, implicating that HupA decreased neuronal death in a diabetic rat model. Consistently, it was previously illustrated that HupA attenuated caspase-dependent neuronal apoptosis in the hippocampus of rats during acute hypobaric hypoxia [31]. Moreover, HupA treatment also ameliorated serum deprivation-induced apoptosis via inhibiting mitochondria-dependent caspase-3 activity in cultures of rat cortical neurons [32].

## 4. Experimental Section

### 4.1. Animals

Male Wistar rats, weighing 220–250 g were selected in the present study. They were kept under a standard environment (12:12 h day/night cycle, 50%–70% humidity, 24 °C) with free access to water and rodent chow. Great efforts were made to minimize the suffering of animals. All experiments conformed to guidelines established by the Ministry of Health, PR China and approved by the Animal Care Committee of Central South University (Changsha, China).

### 4.2. Chemicals and Drugs

HupA (a colorless powder with a purity >99%) and STZ were acquired from Sigma (St. Louis, MO, USA). A glucose oxidase peroxidase diagnostic enzyme kit was supplied from Span Diagnostic Chemicals, India. BDNF E_max_^®^ Immunoassay System and Reverse Transcription System were obtained from Promega (Madison, WI, USA). TRIzol reagent was acquired from Invitrogen (Carlsbad, CA, USA). All other chemicals were of analytical grade.

### 4.3. Induction and Assessment of Diabetes

A single dose of 65 mg/kg STZ dissolved in citrate buffer (pH 4.4, 0.1 M) was injected intraperitoneally to induce diabetes. The age-matched normal rats were treated with an equal volume of citrate buffer only. Development of diabetes was confirmed after 48 h of STZ injection and the plasma glucose levels were determined using an enzymatic glucose oxidase peroxidase diagnostic kit. The rats with fasting plasma glucose levels more than 250 mg/dL [33] were considered to be diabetic animals and selected for the present investigation. Animals in each experiment were randomly assigned to four groups: (1) control group (Con) (*n* = 8), with normal rats that received saline intraperitoneally (physiological saline 0.1 mL/100 g); (2) vehicle group (DM) (*n* = 8), the diabetic rats that received saline intraperitoneally (physiological saline 0.1 mL/100 g); (3,4) HupA groups (DM + HupA (0.05) and DM + HupA (0.1)) (*n* = 8), diabetic rats treated with HupA at doses of 0.05 and 0.1 mg/kg, respectively. The dosage and dosing frequency of HupA were selected according to the previous reports [18,31]. HupA was freshly prepared by dissolving in the physical saline and injected intraperitoneally once a day. Starting from the third day of experiment until seventh week, the control and diabetic control groups received vehicle of HupA.

### 4.4. Morris Water Maze Test

Learning and memory performance was assessed by the Morris water maze test as previously described [34]. In brief, after HupA treatment for seven weeks, the Morris water maze was conducted in a circular water tank 90 cm in diameter and 50-cm in height, equipped with a digital pick-up camera 180 cm above the water surface. The pool water temperature was maintained at approximately 25 ± 2 °C and non-toxic white paint was used to make the water camouflaged. Throughout the study, the tank was constantly placed in a visible position and conceptually divided into four equal quadrants, namely N (north), S (south), E (east) and W (west). A round escape platform was submerged 2 cm underneath the water surface. The rats experienced three consecutive daily training trials for 4 days, with an inter-trial interval of 30 min. In each trial, the escape latency (s) and path length (cm) to find the platform were analyzed and averaged over three trials for each rat. Swimming speed was measured by dividing the path length by the time to find the platform. On the fifth day, the escape platform was removed and each rat was subjected to a spatial probe test. The number of times the rat crossed the target quadrant (where the platform was once hidden) and the time spent in the former platform quadrant were measured within 60 s.

### 4.5. Detection of Neuronal Loss in Hippocampus by Nissl Staining

After seven weeks of HupA treatment, animals were transcardially perfused with 4% paraformaldehyde in 0.1 M phosphate buffer (PB). Then brains were quickly dissected and frozen sections (8 μm) were collected by microtome (Leica CM1900 UV, Leica, Solms, Germany). The slides were infiltrated in 0.1% cresyl violet for 10 min at room temperature. Sections were then dehydrated in graded alcohol, coverslipped with neutral balsam and observed under a light microscope. Neurons with round and palely stained nuclei were considered to be viable.

### 4.6. Measurements of Acetylcholinesterase (AChE) and Choline Acetylase (ChAT) Activities

The activities of AChE and ChAT in the cortex and hippocampus from different groups were measured by colorimetry according to the corresponding commercial kits (kit No. A023 for AChE and kit No. A079-1 for ChAT, Nanjing Jiancheng Biotechnology Institute, Nanjing, China). The absorbance was recorded at 532 and 324 nm, respectively.

### 4.7. RNA Extraction and Quantitative Real-Time RT-PCR Analysis

The total RNA was extracted from cerebral cortex and hippocampus samples using the Trizol method (Invitrogen Technology, Carlsbad, CA, USA) as directed by the manufacturer’s protocol. Primer sequences used for real-time RT-PCR analysis were summarized in Table 2. Equal amounts of RNA (500 ng) was reversely transcribed into complementary DNA using Takara RNA PCR Kit (AMV) Ver.3.0 (TaKaRa Bio Inc., Tokyo, Japan). Afterwards, SYBR Green I-based fluorescent detection was performed by ABI PRISM 7500 with the following thermal conditions: preincubation at 95 °C for 30 s, followed by 45 cycles (denaturation at 95 °C for 10 s and renaturation at 62 °C for 31 s). β-actin was considered as a housekeeping gene. The results were represented as a ratio: BDNF/β-actin mRNA.

### 4.8. Determination of BDNF Content

After the behavioral test, all the animals were sacrificed immediately and the cerebral cortex and hippocampus were carefully dissected. These samples were homogenized in an ice-cold lysis buffer (HEPES 25 mM, MgCl_2_·6H_2_O 5 mM, EDTA·2Na, 5 mM, pH 7.4, 0.5% (*v*/*w*) Triton X-100, DTT 5 mM, PMSF 2 mM, Pepstation A 10 μg/mL and Leupetion 10 μg/mL). After centrifugation at 10,000× *g* for 10 min, the supernatants were collected and total protein concentration was determined by Coomassie blue method. BDNF content in cerebral cortex and hippocampus was analyzed using BDNF Emax^®^ Immunoassay system (Madison, WI, USA) following the manufacture’s protocols.

### 4.9. Assessment of Oxidative Stress

After HupA treatment, the specific markers for oxidative stress including malondialdehyde (MDA), superoxide dismutase (SOD), glutathione peroxidase (GSH-PX) and catalase (CAT) in cerebral cortex and hippocampus tissues were analyzed by commercial kits (Nanjing Jiancheng Biotechnology Institute, Nanjing, China).

### 4.10. Measurements of Inflammatory Cytokines

The p65 subunit has a positive correlation with activated NF-κB signaling. The activities of NF-κB p65, TNF-α, IL-1β, IL-6 and IL-10 in cerebral cortex and hippocampus were measured by ELISA using respective immunoassay kits (Catalog No. H202 for NF-κB, Nanjing Jiancheng Biotechnology Institute, Nanjing, China; No. RTA00 for TNF-α, No. SRLB00 for IL-1β, No. R6000B for IL-6 and No. R1000 for IL-10, R&D Systems, Minneapolis, MN, USA) according to the manufacturer’s protocols.

### 4.11. Measurements of Caspase-3 Activity

Caspase-3 activity was determined by colorimetric method according to the manufacturer’s instructions (Catalog No. BF3100, R&D Systems, Minneapolis, MN, USA). The absorbance was recorded at 405 nm.

### 4.12. Statistics

All results were represented as mean ± S.D. The data was analyzed by SPSS 16.0 software. Statistical significance was determined using one-way ANOVA followed by Dunnett’s test and a *p* value of less than 0.05 was deemed statistically significant.

## 5. Conclusions

Taken together, it is concluded that HupA exerts beneficial effects on blood glucose, learning and memory functions, and neuronal damage; its neuroprotection may be associated with elevating BDNF expression, decreasing oxidative stress, inhibiting the NF-κB signaling pathway and suppressing caspase-3 activity in diabetic rats. These results point toward the potential of HupA as a drug for the treatment of conventional anti-hyperglycemic regimens as well as DACD, but further investigations should be carried out.

## Figures and Tables

**Table 1.** Effect of HupA on body weight and blood glucose levels (*n* = 8, mean ± S.D.) in the four groups of rats at the onset and at the end of the experiment.

Treatment	Body weight (g)		Plasma glucose (mg/dL)	
	
	Onset of study	End of study	Onset of study	End of study
Con	242.20 ± 4.38	285.70 ± 4.27	114.30 ± 2.57	108.00 ± 1.53
DM	245.23 ± 5.31	140.21 ± 4.89 **	110.14 ± 2.62	585.10 ± 3.68 **
DM + Hup (0.05)	239.90 ± 5.36	232.50 ± 3.28 ##	106.25 ± 2.79	306.90 ± 3.87 ##
DM + HupA (0.1)	240.50 ± 5.43	256.40 ± 5.25 ##	107.48 ± 2.39	299.00 ± 3.48 ##

** *p* < 0.01 compared with Con group;

## *p* < 0.01 compared with DM group.

Con, control; DM, diabetes; DM + HupA (0.05), huperzine A (0.05 mg/kg)-treated; DM + HupA (0.1), huperzine A (0.1 mg/kg)-treated groups.

**Table 2.** Primer sequences used in real-time RT-PCR.

Gene	Primer sequences(5′-to-3′)	PCR product size	Accession number
BDNF	Forward:5′- ATGGGTTACACGAAGGAAGG -3′ Reverse:5′- CCGAACATACGATTGGGTAGT -3′	84 bp	NM_012513.3
β-actin	Forward:5′- AGGCCCCTCTGAACCCTAAG -3′ Reverse:5′- CCAGAGGCATACAGGGACAAC -3′	118 bp	EF156276
