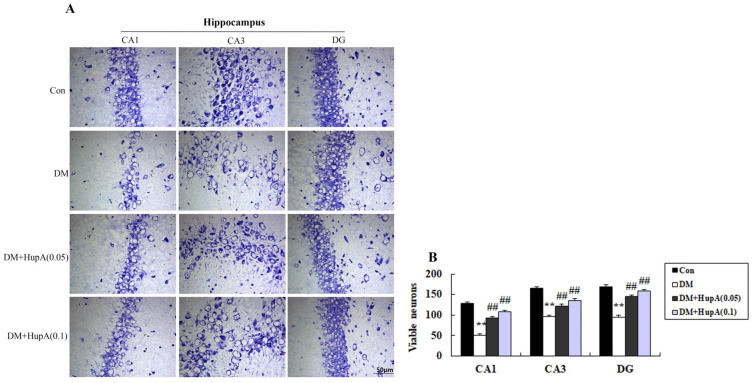# Correction: Mao et al. Huperzine A Ameliorates Cognitive Deficits in Streptozotocin-Induced Diabetic Rats. *Int. J. Mol. Sci*. 2014, *15*, 7667–7683

**DOI:** 10.3390/ijms26125746

**Published:** 2025-06-16

**Authors:** Xiao-Yuan Mao, Dan-Feng Cao, Xi Li, Ji-Ye Yin, Zhi-Bin Wang, Ying Zhang, Chen-Xue Mao, Hong-Hao Zhou, Zhao-Qian Liu

**Affiliations:** 1 Institute of Clinical Pharmacology, Hunan Key Laboratory of Pharmacogenetics, Central South University, Changsha 410078, China; maoxiaoyuan2011@163.com (X.-Y.M.); xilidr@163.com (X.L.); jiyeyindr2014@163.com (J.-Y.Y.); chenxuemao2014@163.com (C.-X.M.); honghaozhoudr@163.com (H.-H.Z.); 2 Institute of Clinical Pharmacology, Xiangya Hospital, Central South University, Changsha 410008, China; 3 Department of Genetics, Institute of Medical Biology, Chinese Academy of Medical Sciences & Peking Union Medical College, Kunming 650118, China; danfengcao2014@163.com; 4 School of Life Sciences, Zhengzhou University, Zhengzhou 450001, China; zhibinwang2014@163.com

The journal corrects the article “Huperzine A Ameliorates Cognitive Deficits in Streptozotocin-Induced Diabetic Rats” [1], cited above.

Following publication, concerns were brought to the attention of the publisher regarding figure duplication within this article [1].

Adhering to our complaints procedure, an investigation was conducted that confirmed the presence of a duplication of two panels contained within Figure 3. The authors cooperated with the Editorial Office during the investigation and provided the original raw material for Editorial Board evaluation. The corrected Figure 3A is attached below.

The authors apologized for the careless mistake and declare again that the conclusion is accurate.

New version of Figure 3A: